# Clinical nurses’ work procrastination and smartphone addiction: a potential profile study

**DOI:** 10.3389/fpsyg.2024.1387288

**Published:** 2024-07-31

**Authors:** Huiyuan Xue, Songbin Jing, Xiaoren Song, Fen Zhang, Xiaoli Liu, Xiaona Si

**Affiliations:** ^1^Department of Neurology, People’s Hospital of Henan University of Traditional Chinese Medicine, Zhengzhou, Henan, China; ^2^Nursing Department, People’s Hospital of Henan University of Traditional Chinese Medicine, Zhengzhou, Henan, China

**Keywords:** procrastination, potential profile analysis, nurse, heterogeneity, smartphone addiction

## Abstract

**Background:**

In the medical field, effective time management by clinical nurses is crucial for enhancing the quality of patient care. However, in recent years, with increasing work pressure for clinical nurses, procrastination has become a prevalent issue. Many nurses use smartphones as a way to alleviate stress and manage emotions, but excessive smartphone use could exacerbate procrastination, thereby jeopardizing patient safety and healthcare quality. Therefore, understanding the current state of work procrastination among clinical nurses, its heterogeneity, and exploring the impact of smartphone addiction and demographic factors on different aspects of nurse procrastination hold significant importance for improving patient care quality.

**Objective:**

This study aims to explore the current state of work procrastination among clinical nurses and identify potential profile categories. It further analyzes the impact of mobile phone addiction and demographic factors on work procrastination among clinical nurses.

**Methods:**

Convenience sampling was employed to recruit participants from three tertiary hospitals in central China from October to November 2023. Surveys measuring nurses’ work procrastination and smartphone addiction were distributed and collected through online platforms. A total of 1,536 nurses participated in this study. Mplus 8.3 statistical software was used for latent profile analysis of clinical nurses’ work procrastination, and SPSS 26.0 software was utilized for chi-square tests, rank-sum tests, and multi-classification logistic regression analyses.

**Results:**

The median total score for clinical nurses’ work procrastination was 21.00 (17.00, 28.00), and three subgroups were identified: low procrastination (66.93%), medium-low procrastination (20.66%), and medium-high procrastination (12.41%). Additionally, logistic regression analysis revealed that smartphone addiction and department atmosphere were common influencing factors for medium-low and medium-high work procrastination. Hospitals with stricter management and nurses holding the position of head nurse were more likely to belong to the low work procrastination group. Nurses with higher incomes or those holding intermediate titles were more prone to medium-low work procrastination, while those experiencing career advancement difficulties were more likely to exhibit medium-high work procrastination (*p* < 0.05).

**Conclusion:**

Clinical nurses’ work procrastination is generally at a medium-to-low level, with three subgroups identified: low procrastination, medium-low procrastination, and medium-high procrastination. Additionally, clinical nurses in surgical departments or those with intermediate titles exhibit higher levels of procrastination. Factors such as smartphone addiction, higher monthly income, tense departmental atmosphere, and unsuccessful career advancement are more likely to lead to work procrastination. Conversely, nurses in hospitals with strict management or those holding the position of head nurse exhibit lower levels of work procrastination. Therefore, nursing managers should pay close attention to the work procrastination behaviors of clinical nurses, actively monitor predictive factors among different groups, and provide psychological counseling and relevant training based on individual nurse circumstances. Additionally, it is also essential to focus on and improve departmental atmosphere and nurse smartphone addiction to enhance clinical nurses’ work efficiency and reduce work procrastination.

## 1 Introduction

Nowadays, with the advancement of science and technology, the way of human life has undergone significant changes. Innovations such as the internet, smartphones and so on have made human life more convenient and the pace of life is getting faster and faster. However, while technological innovations bring freshness to our lives, they also offer more choices and temptations, making it difficult for us to focus on tasks at hand, leading to distractions and procrastination. Work procrastination refers to the behavior of intentionally delaying the completion of tasks without reasonable reasons (Steel, 2007). As a common behavioral phenomenon, it has been a research focus in the fields of psychology and management (Metin et al., 2016; Sirois et al., 2023). Although work procrastination is widely considered a time management issue at the individual level, recent studies have started to explore its complexity in different occupational environments (Metin et al., 2018). In the rapidly developing modern healthcare system, clinical nurses play a crucial role as one of the largest components of the healthcare team. Their work efficiency directly impacts the quality of patient care. Therefore, the manifestation of work procrastination in the nursing profession has gradually received attention (Basirimoghadam et al., 2023). However, with the increasing intensity of clinical work and the complexity of the practice environment, nurses face growing pressure. Work procrastination, as a prevalent psychological and behavioral issue, has become an important factor affecting the efficiency of clinical nurses (Basirimoghadam et al., 2020; Babaie et al., 2022). Research also indicates that work procrastination not only affects nursing efficiency and quality but may also pose a threat to patient safety, which in turn has a negative impact on nurses’ mental health and professional identity (Basirimoghadam et al., 2023). Additionally, the unique nature of nursing work, including workload, interpersonal conflicts, and the uncertainty of career development, may exacerbate the procrastination tendency among clinical nurses, making them more vulnerable when facing work procrastination (Dai et al., 2019). Therefore, in a complex and diverse healthcare environment, it is increasingly important to pay attention to the work procrastination among clinical nurses.

In recent years, although academic research on the phenomenon of nursing procrastination has gradually increased, exemplified by Babaie et al. (2022) cross-sectional study on 125 Iranian nurses and Basirimoghadam et al.’s (2023) exploration of the correlation between clinical nurse procrastination and self-health. However, the majority of nursing research has predominantly adopted a variable-centric approach, focusing on the interrelations of variables, which has not sufficiently identified the heterogeneity in work procrastination among clinical nurses. In contrast, Latent Profile Analysis (LPA) is a research method that divides the measured objects into several different categories through observable continuous variables and further studies population characteristics through the distribution proportions of categories (Spurk et al., 2020). Different from traditional clustering analysis methods, LPA is human-centered and can identify and describe hidden groups or subgroups in the data, thus providing important complementary information to traditional analysis methods (Kalamara and Richardson, 2022). Due to these advantages, LPA has been widely applied in the fields of sociology, psychology, and medicine. Therefore, this study uses the method of LPA to identify different subtypes and influencing factors of procrastination in clinical nurses’ work. By analyzing the procrastination behavior of clinical nurses, it aims to provide strategic suggestions for nursing managers to better understand and improve the work status of nurses, thereby enhancing the efficiency and quality of the entire nursing team. At the same time, it also provides a new perspective and empirical data support for the relevant theoretical research on procrastination in the field of nursing.

## 2 Background

### 2.1 Work procrastination

In recent years, internet buzzwords such as “mo yu” (slacking off) and “tang ping” (lying flat) have rapidly gained popularity across various industries in China. Upon closer examination, one can discover that the fast-paced lifestyle, high medical and education expenses, and the 996-work culture (working from 9 am to 9 pm, 6 days a week, with at least 10 h of work per day) have imposed heavy work pressures on people. Despite the saying that pressure leads to motivation, excessive pressure can have negative effects. Due to the human nature to seek pleasure and avoid harm, people are more inclined to choose easy work or lifestyle. This is why phenomena like “mo yu” and “tang ping” which involve procrastination in work occur (Wang et al., 2021). Work procrastination refers to the deliberate delay of tasks in the work environment and the display of distractions unrelated to the work (Metin et al., 2016). The term “procrastination,” as a well-known and slightly derogatory word, originates from the Latin word “Prorastinare,” in which “Pro” means “forward” and “Castinus” refers to “tomorrow.” It initially appeared as a neutral term, but gradually gained negative connotations after the Industrial Revolution in the mid-18th century (Steel, 2007). Research on procrastination dates back to 3,000 years ago, with the earliest written record found in the ancient Greek poet Hesiod’s epic poem “Works and Days.” The most well-known quote is from the British Earl Lord Chesterfield: “No idleness, no laziness, no procrastination; Never put off till tomorrow what you can do today” (Steel, 2007). In addition, the poem “Song of Tomorrow” by Qian Hetan, a poet of the Ming Dynasty in ancient China, it is also written that “Tomorrow comes again and again, and tomorrows are so many. Everyday I wait for tomorrow, and everything comes to be in vain,” which also reflects the exploration of procrastination by ancient people. In modern times, research on procrastination began with the article “Overcoming Procrastination” published by scholars Knaus and William in 1973, and since then the research on procrastination has continued to emerge (Steel, 2007). Today, studies on work procrastination have been comprehensively summarized and have delved into various fields.

Based on the theory of temporal motivation, it can be seen that work procrastination is a consideration after individual psychological balance. The question that “Do it now or later?” is not only the core issue of the psychological mechanism of work procrastination but also a decision-making problem. Individuals with lower subjective sensitivity to work procrastination are more prone to procrastination (Zhang et al., 2019). People are more willing to engage in tasks with high expectations and value, leading to the occurrence of phenomena such as “browsing the internet during work,” “slacking off,” and “getting busier as procrastination continues”(Metin et al., 2016; Lim and Teo, 2024). However, for modern enterprises, time is considered a scarce resource. Employees who can better organize their time are considered more valuable than those with poorer time management skills (Zhang et al., 2019). Only when each employee is diligent, efficient and engaged in work can the normal operation of the interest chain can be realized, and the employee’s procrastination behavior is bound to cause the reduction of individual labor output (Hen et al., 2021). Therefore, as a result of the failure of self-regulation by employees, work procrastination not only affects their physical and mental health but also has a serious impact on the overall work process (Rebetez et al., 2018; Chen et al., 2020). As an important part of the modern healthcare system, nursing is crucial for the health and safety of patients. However, in the current complex healthcare landscape, various factors such as high stress levels, unreasonable workloads, and interpersonal conflicts within the work environment may induce clinical nurses to engage in procrastination as a temporary escape mechanism. Empirical studies also have corroborated the prevalence of procrastination behaviors among nurses, with demographic variations such as age, marital status, and professional titles emerging as significant antecedents influencing work procrastination (Ma et al., 2021; Babaie et al., 2022). It is noteworthy that most studies on nurse work procrastination have adopted a variable-centered approach, reflecting the overall procrastination situation through surveys. However, due to individual differences, the heterogeneity of work procrastination among nurses has not been adequately identified. Consequently, there remains a necessity to conduct latent profile analyses of clinical nurse work procrastination and to explore the impact of demographic differences on these profiles, thereby providing reference for mitigating nurse work procrastination levels. Based on the foregoing discussion, the following hypotheses are posited for this study:

*Hypothesis 1*: There are different subgroups of clinical nurses’ work procrastination behavior of clinical nurses.

*Hypothesis 2*: There are differences among different demographic clinical nurses in their work procrastination subgroups.

### 2.2 Smartphone addiction

Since the 21st century, information science and technology have pushed society into a new era of digital networking. The emergence of the Internet and mobile phones has made human life more convenient, profoundly changing the way people live and work (Ratan et al., 2021; Lian et al., 2022). As one of the most important products of the digital age, smartphones have gradually become indispensable in society. Since the entry of the iPhone into the market in 2007, the development of smartphones has entered a stage of rapid development, and the number of smartphone users has grown exponentially (Elhai et al., 2017). According to App Annie’s “State of Mobile 2022” report, 3.8 trillion hours of mobile time are used worldwide (Olson et al., 2022). With the rapid development of technology, smartphone functions have become increasingly diverse, permeating every aspect of life from communication and entertainment to information retrieval and personal management. In the palm of one’s hand, it undertakes the vast world, allowing people to know about the world without leaving their homes. However, despite the unprecedented convenience provided by these compact and powerful devices, they have also brought about some potential issues, with smartphone addiction being the most concerning (Olson et al., 2023). Smartphone addiction, also known as smartphone dependency, refers to individuals losing control over their use of smartphones, to the extent that it affects daily life, work, and interpersonal relationships (Osorio-Molina et al., 2021). Its characteristics include excessive reliance, compulsive checking, prolonged usage, and feelings of anxiety and loss when without the phone (Brand et al., 2019). Although it has not been formally included in diagnostic manuals for mental disorders, such as the “Diagnostic and Statistical Manual of Mental Disorders” (DSM-5), it has been increasingly defined and quantified by psychologists and sociologists (Panova and Carbonell, 2018; James et al., 2023; Lin et al., 2023). Related studies have also indicated that the rate of smartphone addiction is as high as 38% in the population, and behaviors like not being separated from the smartphone, frequently checking it, and “phubbing” have become new social phenomena (Karadağ et al., 2015; Luk et al., 2018). However, it is worth noting that although smartphones have changed human life, the harm of smartphone addiction cannot be ignored. Research has shown that prolonged use of smartphones can lead to neck and back pain, vision and psychological problems, as well as degradation of social skills, causing feelings of loneliness and social disorders (Benites-Zapata et al., 2021; Ratan et al., 2021; Schroeder et al., 2022). Moreover, studies on the neurological mechanisms also confirm that addictive behavior can excessively activate the human prefrontal dopamine system, making individuals disinterested in other activities, leading to behaviors such as distraction and rigidity (Volkow et al., 2017; Darnai et al., 2019).

However, smartphones, as intelligent mobile devices, are now widely used in medical environments. As an important member of the medical team, clinical nurses are inevitably affected by smartphones in today’s paperless and electronic office medical system (De Jong et al., 2020). At the same time, due to the particularity of the nature of the work of clinical nurses, high-intensity work pressure and emotional labor may prompt them to seek smartphones as a way to relieve stress and emotions (Ma et al., 2021). Based on the theory of use and gratification, smartphones have become a habitat and foothold for individual expectations due to their convenience and the new stimulus information they carry, which can meet the specific psychological needs and pleasant experiences of clinical nurses to a certain extent (Yu, 2024). However, excessive reliance on smartphones will inevitably lead to distraction and procrastination in nursing work, which in turn affects their work efficiency and professional performance (Buneviciene and Bunevicius, 2021). Pertinent research indicates that over half of nurses engage in non-work-related activities on their mobile phones during work hours, which negatively impacts their work efficiency and professional performance (Mcbride et al., 2015). Moreover, with the advent of the AI era, digitalization and intelligence are poised to become new frontiers in nursing development. Smartphones, as pivotal platforms for data, will increasingly intersect with clinical nurses’ daily routines, potentially intensifying their reliance on these devices and leading to smartphone addiction. This, in turn, could adversely affect work efficiency and contribute to procrastination (Bautista, 2020; O'connor et al., 2020). Therefore, proactive attention to the current state of clinical nurses’ smartphone addiction and its relationship with procrastination behaviors is not only beneficial for enhancing nurses’ physical and mental well-being but also crucial for elevating nursing quality. Based on the above arguments, this study proposes the following hypotheses:

*Hypothesis 3*: There is a positive correlation between clinical nurses’ smartphone addiction and their work procrastination.

## 3 Materials and methods

### 3.1 Study design and participants

Convenient sampling was employed for participant recruitment in three tertiary hospitals in central China from October to November 2023. Inclusion criteria were as follows: (1) Registered nurses in the People’s Republic of China; (2) Informed and voluntary participation in the study. Exclusion criteria included: Intern nurses or nurses who had been away from their positions for 3 months due to other reasons. The distribution and collection of the survey questionnaire were conducted through the internet platform^1^. Prior to distributing the questionnaire, approval was obtained from the head of the nursing department at each participating hospital. With their assistance, the questionnaire was distributed to various departments. The questionnaire was filled out anonymously, and the purpose, filling method, and precautions were uniformly explained on the questionnaire homepage. The data collection period spanned 2 months, with the questionnaire being distributed twice—once in early October and again in early November. Meanwhile, to avoid data bias, the number of times each mobile IP address could fill out the questionnaire was limited to once. The questionnaires with inconsistent answers, consistent self-assessment scores for all items, or completion times less than 60 s were excluded.

This study is a quantitative cross-sectional research conducted in strict adherence to the STROBE guidelines. The sample size calculation was based on the 10 events per variable (10 EPV) principle and a general generalized multivariate analysis design, i.e., the sample size was 5–10 times the total number of questionnaire items (Riley et al., 2020). Therefore, the sample size for this study is: *N* = (16 + 12 + 17) *10 = 450. Additionally, a 20% expansion was applied to the original sample size to avoid data loss due to invalid questionnaires. Consequently, a minimum of 540 nurses needed to be included. In this study, a total of 1,536 nurses participated, with 1,418 valid questionnaires, resulting in an effective response rate of 92.32%. The participants had an average age of 34.13 years (IQR 34.00, range 20 ~ 59 years), with the majority being female. Most participants held a bachelor’s degree (88.08%), and 72.07% of the nurses were married.

### 3.2 Ethics statement

This study was conducted under the ethical guidelines of the “Helsinki Declaration,” with the survey being filled out anonymously, and all participating nurses provided informed consent. The study received ethical approval from the Ethics Committee of People’s Hospital of Henan University of Traditional Chinese Medicine, the institution with which the researcher is affiliated, in September 2023 (Ethical Approval Number: 2023091152).

### 3.3 Measures

#### 3.3.1 Demographic

The baseline data of the participants are designed by the researchers based on relevant information. There were 13 items in total, including age (years), gender (Male, Female), education levels (Junior college, Undergraduate, Master degree or above), marital status (Single, Married, Widowed, or separated), number of children (0, 1, ≥2), working years (≤5 years, 6 ~ 10 years, 11 ~ 15 years, >15 years), department (Internal Medicine, Surgical, Obstetrics and Gynecology, Pediatrics, Emergency, Intensive Care Unit and Operating Room, Outpatient and others), job title (Primary title, Intermediate title, Senior title), positions (Head nurse, Nurse), monthly income [(Renminbi, RMB) <5,000, 5,000 ~ 8,000, >8,000], department atmosphere (Disharmonious, generally, Harmonious, Very harmonious), promotion of professional titles (Not smooth, generally, smoothly), and hospital management (Very strict, Strict, generally).

#### 3.3.2 Procrastination at work scale (PAWS-C)

The scale was translated into Chinese by Wang et al. (2021) based on the Chinese translation of the Work Procrastination Scale developed by Metin et al. (2016). It is mainly used to assess the work procrastination of employees in the context of Chinese culture (Metin et al., 2016; Wang et al., 2021). The scale consists of two dimensions: “loafing at work” and “cyber loafing.” The dimension of loafing at work is composed of 8 items, such as “Even if I make a plan at work, I still delay its execution” and “When work tasks are boring, I tend to daydream and find it difficult to focus.” The dimension of cyber loafing includes 4 items, such as “During work hours, I spend more than half an hour on social networking sites.” The scale uses Likert’s 5-point scoring method, where “1 point” to “5 points” represent the range of choices from “never” to “always.” The total score is 60 points, and the degree of work procrastination is directly proportional to the total score. In this study, the Cronbach’s *α* coefficient of the scale was 0.906, with Cronbach’s *α* coefficients of 0.890 for loafing at work and 0.888 for cyber loafing, respectively.

#### 3.3.3 Mobile phone addiction index (MAPI)

The MAPI scale was developed by the Hong Kong scholar Leng and has good applicability in the Chinese-speaking region (Leung, 2008). The scale consists of four dimensions: inability to control carving (e.g., Your friends and family complained about your use of the mobile phone), feeling anxious and lost (e.g., You feel lost without your mobile phone), withdrawal or escape (e.g., You have used your mobile phone to make yourself feel better when you were feeling down), and productivity loss (e.g., Your productivity has decreased as a direct result of the time you spend on the mobile phone), comprising a total of 17 items. Each item is scored on a scale from 1 (never) to 5 (always), with higher scores indicating more severe mobile phone addiction. In this study, the Cronbach’s *α* coefficient of the scale was 0.908, and the Cronbach’s *α* coefficients for the four dimensions ranged from 0.849 to 0.883.

### 3.4 Statistical analysis

IBM SPSS Statistics 26.0 and Mplus 8.3 software are used for data analysis, which was divided into three parts. Firstly, Mplus was used to perform latent profile analysis on clinical nurse procrastination. The fit evaluation indicators were Akaike Information Criterion (AIC), Bayesian Information Criterion (BIC), and Sample Size-Adjusted BIC (aBIC). Smaller values of these indicators indicate a better fit. Likelihood Ratio Test (LRT) and Bootstrapped Likelihood Ratio Test (BLRT) were used to compare the differences between the Kth model and the K-1th model, with a significance level of *p* < 0.05 indicating that the Kth model is superior. Entropy value was used to represent the accuracy of classification, with values closer to 1 indicating more accurate classification (Nylund-Gibson et al., 2019).

Secondly, SPSS is used to perform rank-sum or chi-square tests to analyze whether nurses of different profile categories have demographic differences. Kruskal–Wallis one-way ANOVA test is then used to compare the scores of job procrastination and smartphone addiction among different types of nurses.

Finally, variables with statistically significant differences in the single-factor analysis are included in the regression analysis and the multivariate analysis of different latent profile classifications of clinical nurses’ work procrastination was performed using multivariate logistic regression to explore the factors affecting category attribution. Meanwhile, in this study, descriptive statistics such as frequency and composition ratio are used for count data, and non-normally distributed metric data are represented by IQR [*M* (*P*_25_, *P*_75_)]. Common method bias detection and questionnaire reliability testing are analyzed by SPSS. *α* is set as two-tailed, with *p* < 0.05 indicating statistically significant differences.

## 4 Results

### 4.1 Common method bias detection

According to the suggestion of Podsakoff and Organ (1986), exploratory analysis was conducted on the unrotated principal component factors of the project. The results show that the interpretation rate of the first principal component factor is 16.945%, which has not yet reached half of the total interpretation rate, indicating that there is no serious common method bias in the data.

### 4.2 Current status and potential profile classification of clinical nurses’ work procrastination

In this study, the median total score of work procrastination for 1,418 nurses was 21.00 (17.00, 28.00), indicating that the level of work procrastination among clinical nurses is moderately low.

**Figure 1:**
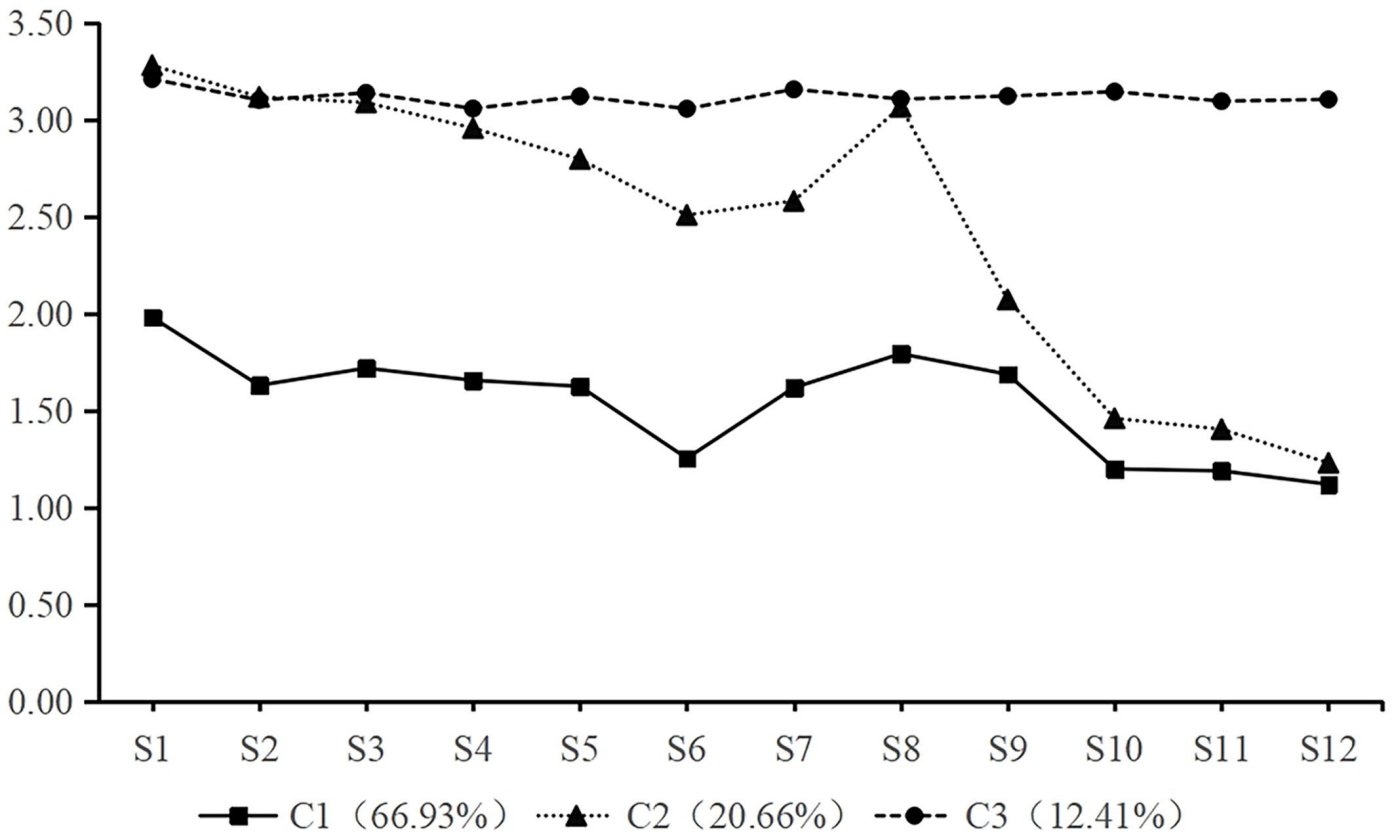
Trend graph of the three categories score from the clinical nurse work procrastination scale.

In addition, four potential profile models were extracted in the study, (see Table 1). It can be observed that with the increase of model classification, the fitting indices AIC, BIC, and aBIC gradually decrease, and the Entropy value gradually increases. However, when the profile model is classified into four categories, the LMRT value is 0.3072, indicating no statistically significant difference compared to the 3-category model. Therefore, based on the fitting indices and practical significance, this study chose the 3-category latent profile model. At the same time, from the category attribution matrix in Table 2, it can be seen that the average probability of each profile category (row) belonging to each column is greater than 90%, indicating that the 3-category model is highly credible. Based on this, the study further obtained the response probability graph of clinical nurses on the Work Procrastination Scale’s 12 items when using the 3-category latent profile, where items S1 ~ S8 represent “delaying work,” and S9 ~ S12 represent “browsing the internet at work,” as shown in Figure 1.

**Table 1:** Fit indices for the latent profile analysis of clinical nurse work procrastination.

Model	AIC	BIC	aBIC	Entropy	LMRT	BLRT	Categorical probability (%)
1	46174.832	46301.000	46224.761	—	—	—	1
2	40239.520	40434.030	40316.494	0.950	0.9441	0.0000	76.38/23.62
3	37730.929	37993.779	37834.947	0.948	0.0000	0.0000	66.93/20.66/12.41
4	37105.203	37436.394	37236.265	0.933	0.3072	0.0000	58.60/17.91/11.50/11.99

**Table 2:** Matrix of latent profile category attribution for clinical nurse work procrastination.

Category	C1	C2	C3
C1	0.988	0.012	0.000
C2	0.063	0.932	0.005
C3	0.000	0.008	0.992

In addition, it can be observed that the total scores and dimension scores of clinical nurses’ procrastination in the 3-category model increase in the order of C1 < C2 < C3 (see Table 3). Among them, the median score of procrastination in the C1 category is 18.00 (16.00, 21.00) points, and each dimension is also at a lower level, with a total of 949 people, accounting for 66.93%; the total score of the C2 category is 29.00 (26.00, 32.00) points, with 293 people (20.66%); the C3 category has the smallest proportion (12.41%), but the total score is relatively high, at 36.00 (36.00, 38.00) points. Therefore, according to the median score of the 3-category latent profile total scores and the linear trend graph in Figure 1, this study names C1 ~ C3 as “Low procrastination,” “mid-low procrastination,” and “mid-high procrastination,” respectively.

**Table 3:** Comparison of scale scores between different latent profiles in clinical nurses.

Items		Profile category			Comparison between groups	*Z*	*p*
		C1 (*n* = 949)	C2 (*n* = 293)	C3 (*n* = 176)			
Procrastination at work							
	Total score	18.00 (16.00, 21.00)^a, b^	29.00 (26.00, 32.00)^c^	36.00 (36.00, 38.00)	C1<C2<C3	950.654	0.000
	Loafing at work	13.00 (11.00, 16.00)^a, b^	23.00 (21.00, 25.00)	24.00 (24.00, 25.75)	C1<C2<C3	909.247	0.000
	Cyber loafing	5.00 (4.00, 6.00)^a, b^	6.00 (5.00, 8.00)^c^	12.00 (12.00, 12.00)	C1<C2<C3	548.165	0.000
Smartphone addiction							
	Total score	33.00 (26.00, 39.00)^a, b^	42.00 (33.50, 49.00)^c^	51.00 (51.00, 51.00)	C1<C2<C3	393.866	0.000
	Inability to control carving	11.00 (8.00, 13.00)^a, b^	14.00 (11.00, 17.00)^c^	21.00 (21.00, 21.00)	C1<C2<C3	443.887	0.000
	Feeling anxious and lost	12.00 (9.00, 16.00)^a, b^	15.00 (11.00, 19.00)	15.00 (15.00, 15.00)	C1<C2<C3	96.970	0.000
	Withdrawal or escape	6.00 (4.00, 8.00)^a, b^	8.00 (6.00, 9.00)^c^	9.00 (9.00, 9.00)	C1<C2<C3	206.194	0.000
	Productivity loss	3.00 (2.00, 4.00)^a, b^	5.00 (4.00, 6.00)^c^	6.00 (6.00, 6.00)	C1<C2<C3	434.717	0.000

C1, low procrastination; C2, mid-low procrastination; C3, mid-high procrastination. ^a^Comparison of C1 and C2; ^b^Comparison of C1 and C3; ^c^Comparison of C2 and C3, all at *P* < 0.05, have been adjusted for significance values by Bonferroni correction for multiple tests.

### 4.3 General data and univariate analysis of work procrastination of clinical nurses

The univariate analysis of different demographic variables in the latent profile classification revealed statistically significant differences (*P* < 0.05) in clinical nurses’ age, gender, marital status, number of children, working years, department, job title, position, and monthly income, as shown in Table 4.

**Table 4:** Univariate analysis of the demographic characteristics of clinical nurses across different latent profile categories of work procrastination.

Variables		Number of people	Profile category			*Z*/*χ*^2^	*p*
			C1 (*n* = 949)	C2 (*n* = 293)	C3 (*n* = 176)		
Age (years)		1,418 (100)	34.00 (29.00, 39.00)	34.00 (28.00, 38.00)	32.00 (27.00,37.00)	14.903	0.001^a^
Gender						11.187	0.004
	Male	67 (4.72)	40 (4.21)	10 (3.41)	17 (9.66)		
	Female	1,351 (95.28)	909 (95.79)	283 (96.59)	159 (90.34)		
Education levels						2.379	0.667^b^
	Junior college	164 (11.57)	115 (12.12)	31 (10.58)	18 (10.23)		
	Undergraduate	1,249 (88.08)	832 (87.67)	260 (88.74)	157 (89.20)		
	Master degree or above	5 (0.35)	2 (0.21)	2 (0.68)	1 (0.57)		
Marital status						9.541	0.049^b^
	Single	381 (26.87)	233 (24.55)	86 (29.35)	62 (35.22)		
	Married	1,022 (72.07)	706 (74.40)	204 (69.63)	112 (63.64)		
	Widowed or separated	15 (1.06)	10 (1.05)	3 (1.02)	2 (1.14)		
Number of children						11.465	0.022
	0	481 (33.92)	301 (31.72)	103 (35.15)	77 (43.75)		
	1	503 (35.47)	357 (37.62)	96 (32.77)	50 (28.41)		
	≥2	434 (30.61)	291 (30.66)	94 (32.08)	49 (27.84)		
Working years (years)						28.750	0.000	≤5	334 (23.55)	193 (20.34)	78 (26.62)	63 (35.79)		
	6–10	278 (19.61)	199 (20.97)	51 (17.41)	28 (15.91)		
	11–15	410 (28.91)	272 (28.66)	83 (28.32)	55 (31.25)		
	>15	396 (27.93)	285 (30.03)	81 (27.65)	30 (17.05)		
Department						39.658	0.000
	Internal medicine	424 (29.90)	275 (28.98)	101 (34.47)	48 (27.27)		
	Surgical	222 (15.66)	138 (14.54)	63 (21.50)	21 (11.93)		
	Obstetrics and gynecology	91 (6.42)	75 (7.90)	11 (3.76)	5 (2.84)		
	Pediatrics	37 (2.61)	26 (2.74)	10 (3.41)	1 (0.57)		
	Emergency, intensive care unit and operating room	256 (18.05)	163 (17.18)	45 (15.36)	48 (27.27)		
	Outpatient and other	388 (27.36)	272 (28.66)	63 (21.50)	53 (30.12)		
Job title						11.072	0.026
	Primary title	717 (50.56)	476 (50.16)	134 (45.73)	107 (60.79)		
	Intermediate title	612 (43.16)	411 (43.31)	138 (47.10)	63 (35.80)		
	Senior title	89 (6.28)	62 (6.53)	21 (7.17)	6 (3.41)		
Positions						10.396	0.006
	Head nurse	200 (14.10)	134 (14.12)	53 (18.09)	13 (7.39)		
	Nurse	1,218 (85.90)	815 (85.88)	240 (81.91)	163 (92.61)		
Monthly income (RMB)						22.574	0.000	<5,000	484 (34.13)	337 (35.51)	75 (25.60)	72 (40.91)		
	5,000–8,000	785 (55.36)	527 (55.53)	170 (58.02)	88 (50.00)		
	>8,000	149 (10.51)	85 (8.96)	48 (16.38)	16 (9.09)		
Department atmosphere						24.998	0.000	Disharmonious	9 (0.64)	3 (0.32)	5 (1.71)	1 (0.57)		
	Generally	161 (11.35)	90 (9.48)	41 (13.99)	30 (17.05)		
	Harmonious	569 (40.13)	377 (39.73)	131 (44.71)	61 (34.65)		
	Very harmonious	679 (47.88)	479 (50.47)	116 (39.59)	84 (47.73)		
Promotion of professional title						26.299	0.000	Not smooth	303 (21.37)	182 (19.18)	73 (24.91)	48 (27.27)		
	Generally	641 (45.20)	418 (44.05)	127 (43.35)	96 (54.55)		
	Smoothly	474 (33.43)	349 (36.77)	93 (31.74)	32 (18.18)		
Hospital management						17.265	0.002	Very strict	699 (49.30)	481 (50.68)	138 (47.10)	80 (45.45)		
	Strict	635 (44.78)	424 (44.68)	137 (46.76)	74 (42.05)		
	Generally	84 (5.92)	44 (4.64)	18 (6.14)	22 (12.50)		

^a^For the Kruskal–Wallis *H* test; ^b^likelihood ratio chi-square, the rest are Pearson chi-square, non-normally distributed metric data are represented by the Interquartile Range (IQR), and count data are represented by the composition ratio (%).

### 4.4 Multivariate logistic regression analysis of latent profile categories of clinical nurses’ work procrastination

Multivariate logistic regression analysis is used to analyze the latent profile of clinical nurses’ work procrastination. Among them, C1 (low procrastination), C2 (mid-low procrastination), and C3 (mid-high procrastination) are assigned values of 1, 2, and 3, respectively. Variables with statistical differences in univariate analysis are included in the regression analysis, and the variable assignments can be found in Table 5. The result of the final regression analysis shows that smartphone addiction, department, technical title, department atmosphere, monthly income, position, career advancement, and hospital management strictness are the main influencing factors for the classification of procrastination profiles in clinical nursing work (*p* < 0.05), as shown in Table 6.

**Table 5:** Multivariate logistic regression independent variables assignment.

Variables	Independent variable assignment
Age (years)	Substitute the original value
Smartphone addiction	Substitute the original value
Gender	0 = Female; 1 = Male
Marital status	0 = Widowed or separated; 1 = Married; 2 = Single
Number of children	0 ≥ 2; 1 = 1; 2 = 0
Working years (years)	0 > 15; 1 = 11 ~ 15; 2 = 6 ~ 10; 3 ≤ 5
Department	0 = Internal Medicine; 1 = Surgical; 2 = Obstetrics and Gynecology; 3 = Pediatrics; 4 = Emergency, Intensive Care Unit and Operating Room; 5 = Outpatient and other
Job title	0 = Senior title; 1 = Intermediate title; 2 = Primary title
Positions	0 = Head nurse; 2 = Nurse
Monthly income (RMB)	0 > 8,000; 1 = 5,000 ~ 8,000; 2 < 5,000
Department atmosphere	0 = Disharmonious; 1 = generally; 2 = Harmonious; 3 = Very harmonious
Promotion of professional title	0 = Not smooth; 1 = generally; 2 = smoothly
Hospital management	0 = Very strict; 1 = Strict; 2 = generally

**Table 6:** Multivariate logistic regression analysis of potential profiles of procrastination in clinical nursing work.

Comparison of categories	Independent variable	*β*	*SE*	Wald *χ*^2^	*P*	OR (95%CI)
C2 vs. C1						
	Intercept	−3.386	0.888	14.534	0.000	—
	Smartphone addiction	0.079	0.008	105.955	0.000	1.082 (1.066 ~ 1.099)
	Surgical	0.560	0.229	5.992	0.014	1.750 (1.118 ~ 2.740)
	Intermediate title	0.527	0.232	5.140	0.023	1.694 (1.074 ~ 2.670)
	Disharmonious (Department atmosphere)	2.295	0.867	7.002	0.008	9.928 (1.814 ~ 54.354)
	Generally (Department atmosphere)	0.513	0.250	4.202	0.040	1.671 (1.023 ~ 2.729)
	>8,000 RMB	0.713	0.262	7.400	0.007	2.041 (1.221 ~ 3.412)
	5,000 ~ 8,000 RMB	0.349	0.175	3.965	0.046	1.417 (1.005 ~ 1.998)
C3 vs. C1						
	Intercept	−7.827	1.326	34.865	0.000	—
	Smartphone addiction	0.186	0.012	222.493	0.000	1.205 (1.176 ~ 1.235)
	Harmonious (Department atmosphere)	−0.469	0.239	3.838	0.050	0.626 (0.391 ~ 1.000)
	Head nurse	−1.082	0.447	5.875	0.015	0.339 (0.141 ~ 0.813)
	Not smooth (Promotion of professional title)	0.871	0.322	7.308	0.007	2.390 (1.271 ~ 4.496)
	generally (Promotion of professional title)	0.643	0.277	5.401	0.020	1.902 (1.106 ~ 3.270)
	Very strict (Hospital management)	−1.330	0.416	10.246	0.001	0.264 (0.117 ~ 0.597)
	Strict (Hospital management)	−1.196	0.408	8.573	0.003	0.302 (0.136 ~ 0.673)

C1, low procrastination; C2, mid-low procrastination; C3, mid-high procrastination.

## 5 Discussion

### 5.1 Status quo of clinical nurses’ work procrastination and characteristics of latent profile categories

Procrastination at work is an irrational behavior that involves unjustifiably delaying one’s official duties and is a negative outcome of individual behavior and self-regulation failure (Steel, 2007; Metin et al., 2016). Studies have shown that for clinical nurses, who play a crucial role in caring for and assisting patients, procrastination not only leads to professional burnout, reduced work efficiency, and lower quality of care but also directly affects patient treatment and recovery (Babaie et al., 2022). Therefore, it is essential to actively pay attention to the current state of procrastination among clinical nurses to enhance the efficiency of nursing work. As expected, in this study, an analysis of clinical nurse procrastination based on an individual-centered approach reveals that work procrastination can be classified into three subgroups: low procrastination, moderate-low procrastination, and moderate-high procrastination (Hypothesis 1). The proportion of moderate and above procrastination is 33.07%, indicating heterogeneity in clinical nurse work procrastination and a relatively serious procrastination. At the same time, in this study, the median total score of clinical nurses’ work procrastination is 21 points. Although this is less than half of the total score on the procrastination scale, it still indicates a moderate level of procrastination, higher than the findings of Rezaei et al. (2017) and confirms the severity of clinical nurse work procrastination.

There are some differences between the results of this study and other studies, which may be related to different cultural situations or research tools. Different cultural values or backgrounds may affect the procrastination choices and behavioral explanations of clinical nurses (Basirimoghadam et al., 2020). Additionally, with the increasing demands for nursing quality in China in recent years, nurses are faced with heavy workloads and long working hours, leading to procrastination in their daily tasks. Furthermore, management systems and complex work environments, such as unreasonable work allocation, lack of career advancement opportunities, and tense interpersonal relationships in departments, may reduce the job satisfaction of clinical nurses, resulting in procrastination behavior (Dai et al., 2019). Due to the common psychological tendency of seeking pleasure and avoiding suffering, clinical nurses are more prone to procrastination under high-pressure conditions. At present, the popular term “nei juan” (meaning peers compete to put in more effort to vie for limited resources, leading to a decrease in individual “benefit-to-effort ratio”) in Chinese network vividly illustrates the degree of pressure in various industries in China, and the nursing industry is inevitably involved. Moreover, in the trend changes in Figure 1, item S1 (“Even if I have planned, I will delay the execution”) has the highest score, further indicating the high prevalence of work procrastination among nurses. Therefore, nursing managers should actively pay attention to the workload of clinical nurses, allocate work reasonably, improve the department’s work atmosphere, and provide necessary support and resources. At the same time, procrastination, as an individual behavior and cognitive state, although changing procrastination habits is not an overnight process, effective cognitive training can significantly improve the procrastination behavior of clinical nurses. Therefore, it is also necessary to provide training on time and stress management for clinical nurses to reduce their procrastination levels.

### 5.2 Analysis of the influencing factors of clinical nurses’ work procrastination

The results of this study indicate that, compared to those with low levels of work procrastination, clinical nurses with higher levels of mobile phone addiction are more likely to belong to the middle-low type (OR: 1.082) and middle-high type of work procrastination (OR: 1.205). This further suggests that mobile phone addiction is a risk factor for work procrastination among clinical nurses. With the advent of the digital information age, smartphones have become essential tools for communication and entertainment, largely fulfilling the desire for “having everything at hand.” In the medical field, the occupational characteristics of clinical nurses determine their close connection with smartphones. In the Chinese healthcare environment, clinical nurses work with high work intensity and risks, often needing to deal with more tasks in limited time and facing the double pressures of work and family, which inevitably impacts their psychological well-being (Liu and Aungsuroch, 2019; Hao et al., 2020). Based on the theory of use and gratification, it can be seen that smartphones, as an easy-to-obtain means of escape, effectively satisfy the interpersonal communication and entertainment needs of clinical nurses, and may be overused to relieve stress and anxiety, resulting in addiction (Yu, 2024). Moreover, the overuse of smartphones may further exacerbate clinical nurses’ work procrastination, creating a vicious cycle (Mcbride et al., 2015; Ma et al., 2021). Therefore, despite the necessity and benefits of clinical nurses using phones in certain situations, the use of phones needs to be reasonably controlled. Nursing managers should recognize that improving work efficiency is not only achieved by increasing workload or compressing time but also by actively improving nurses’ mental health and well-being as well as the balance between work and life. Additionally, it is also necessary to do a good job in professional training for clinical nurses, such as providing training in time management and coping with stress, or establishing a good support system to help them better cope with the challenges at work. Nurses themselves should enhance their digital literacy and self-control abilities, and establish healthy phone usage habits, so as to reduce procrastination and improve work efficiency.

Interestingly, surgical nurses were more likely to be classified as middle-low procrastination type in this study, which may be related to the specificity of surgical department. The findings by Cuccia et al. (2022) and Ferramosca et al. (2023) indicate that surgical nurses, who are chronically exposed to various chemical, biological, and physical hazards, often experience higher levels of physical and psychological risk. Moreover, compared to outpatient and other departments, surgical work is characterized by unpredictability and frequent emergencies, requiring nurses to perform tasks under high pressure (Wei et al., 2023). Additionally, the relatively fast-paced nature of surgical nursing, which often involves handling multiple tasks and emergencies such as preparing for surgical procedures, assisting doctors during surgery, and monitoring patients. Prolonged exposure to such high levels of work stress and load may make it difficult for nurses to allocate time effectively between urgent and routine tasks, potentially leading to workplace procrastination (Ferramosca et al., 2023). Therefore, nursing managers should pay close attention to the procrastination psychology of surgical nurses, do a good job in psychological and decompression training, reasonably arrange nurses’ shifts, and implement the scheduling system of matching the new with the old, while nurses themselves need to do their own time management and actively participate in relevant training in the face of the sudden and uncertainty of surgical work, so as to improve the efficiency of clinical work.

Moreover, in the course of their professional careers, nurses who encounter obstacles in career advancement are more prone to workplace procrastination. In the nursing profession, the advancement of professional titles is a significant goal on their career path. Consequently, when they encounter barriers in achieving title advancements, they may experience feelings of frustration and anxiety. Moreover, failure in career progression can lead to nurses questioning their abilities and self-worth, which in turn may trigger procrastination behaviors (Lu et al., 2019). Nursing is inherently a high-stress, high-risk profession, and clinical nurses, who are already under considerable pressure from their work environment, may experience even greater psychological strain when faced with unreasonable career advancement challenges (Si et al., 2023). Therefore, it is imperative for management to make rational arrangements for nurses’ title advancements based on clinical realities, actively provide psychological counseling for those experiencing difficulties in career progression, and for nurses themselves to enhance their professional knowledge and technical skills to improve their core competencies.

In this study, it was found that clinical nurses with intermediate professional titles have a 1.694 times higher risk of experiencing mid-low procrastination compared to nurses with basic professional titles, aligning with the findings of Babaie et al. (2022). This reason may be attributed to the fact that nurses with intermediate titles often serve as key personnel within their departments, possessing more solid clinical experience and knowledge compared to their junior counterparts. Consequently, nursing managers frequently assign them with critical tasks within the department, which can inadvertently impose greater work pressures upon them (Babaie et al., 2022; Wei et al., 2023). Furthermore, due to their higher professional titles, these nurses may be granted greater autonomy and decision-making authority, necessitating the independent handling of more complex cases and situations (Yinghao et al., 2023). In contrast, nurses with entry-level titles are often engaged in more operational tasks with clearer objectives and less managerial responsibility. This difference in work pressure may predispose nurses with intermediate titles to a higher likelihood of engaging in procrastination behaviors (Cuccia et al., 2022). Therefore, it is recommended that nursing managers make good work arrangements for departments, implement humanized management, and adopt flexible scheduling based on the actual situation of the department, trying to better accommodate the needs of clinical nurses at every level and creating a more flexible working environment, so as to reduce the work pressure on clinical nurses ultimately.

Furthermore, the results of this study also suggest that clinical nurses with tense interpersonal relationships in the department are more prone to work procrastination compared to those with harmonious interpersonal relationships, which indicates that the departmental atmosphere is an important influencing factor on clinical nurse work procrastination. Aristotle believed that humans are social beings, no one exists in isolation, and individual activities are inseparable from interaction with other living beings. Maintaining harmonious working relationships among clinical nurses and colleagues can enhance mutual support, making it easier for individuals to carry out clinical work. Good collaboration and mutual respect among department members can create a more positive and comfortable working environment, helping to alleviate the psychological stress of clinical nurses and improve their work efficiency (Si et al., 2023). As demonstrated by Hølge-Hazelton’s and Berthelsen (2020) research, despite the arduous nature of their daily work, the unique culture and harmonious atmosphere within the department foster a closer bond among nurses and mitigate their inclination to leave their positions. Therefore, it is recommended that nursing managers focus on constructing a positive departmental atmosphere, actively paying attention to the interpersonal relationships among nurses, enhance team collaboration, and cultivate a cohesive and friendly professional environment. This approach may help reduce work procrastination among clinical nurses.

At the same time, this study also reveals that nurses with higher monthly income are more likely to engage in work procrastination, which may be related to the work pressure they face. In the Chinese medical environment, generally speaking, the work income of clinical nurses is closely related to the workload of their department. The more patients there are, the faster the turnover of department beds, and the higher the department’s income. However, higher-income nurses may also face greater pressure and responsibility, leading to more frequent procrastination behavior (Wang et al., 2019). Furthermore, according to the theory of motivated behavior (Schunk and Dibenedetto, 2020), individuals’ decision-making can be influenced by irrational factors. High income does not necessarily stimulate greater work motivation; instead, individuals facing high income may experience reduced financial pressure, leading to feelings of complacency, which in turn diminish the sense of urgency toward work and increases procrastination behavior. Therefore, nursing managers should pay greater attention to work procrastination among high-income nurses, optimize work processes to assist nurses in managing time and tasks more effectively, and provide timely psychological health support to enhance their work efficiency.

The results of this study suggest that, compared to regular nurses, head nurse exhibit lower levels of job procrastination. The reason may be that on the one hand, as leaders of the department, nurse managers play a crucial connecting role between the upper leaders of the hospital and the regular nurses, and have strong overall coordination skills in handling various tasks and interpersonal relationships. On the other hand, as leaders of the department, nurse managers need to set a positive example and lead by demonstrating good practices. Compared to regular nurses, they are more likely to prioritize time management, understand the harm caused by procrastination, and therefore exhibit lower levels of procrastination (Morse and Warshawsky, 2021). Additionally, head nurse typically possess higher leadership and responsibility levels, requiring them not only to oversee routine nursing tasks but also to manage teams, coordinate resources, and ensure the efficient operation of healthcare services (Salvage and White, 2019). This leadership and sense of responsibility may prompt them to execute work plans with greater rigor, potentially reducing instances of procrastination.

Finally, in this study, we also found that the degree of clinical nurse job procrastination is negatively correlated with the strictness of hospital management. This may be due to the fact that strict hospital management often implies clearer job requirements and norms, communication channels, and oversight mechanisms, all of which effectively enhance nurses’ sense of responsibility and urgency toward their work, thereby reducing the risk of job procrastination (Genrich et al., 2020). However, it is essential to note that anyone or anything may have a corresponding range of elastic tolerance. According to Self-Determination Theory (SDT), when individuals feel their autonomy is restricted, they may engage in procrastination as a way to seek psychological comfort, and that excessively stringent management may instead lead to nurses developing excessive work pressure and reducing their autonomy (Van Den Broeck et al., 2016). Therefore, the strictness of hospital management should be determined according to the actual situation of clinical nurses. Specific issues require specific analysis to avoid adverse effects.

### 5.3 Limitations

There are still some limitations in this study. Firstly, as a cross-sectional study, the sample data can only represent the situation at a specific point in time, making it difficult to reveal the dynamic evolution of clinical nurses’ procrastination behavior over time, limiting the depth of understanding of this phenomenon. Secondly, in this survey, the data are all self-reported by nurses, which may lead to situations where they conceal their procrastination behavior due to societal expectations or professional ethical considerations. Although we have imposed strict restrictions on questionnaire responses and excluded invalid surveys, there may still be some bias. Furthermore, regarding the sample selection, only nurses from three tertiary hospitals in central China were sampled, which may impose certain regional limitations on the generalizability of the research results. Lastly, due to various factors that may affect clinical nurses’ procrastination, despite incorporating multiple relevant factors in this study, there may still be missing information. Therefore, in future research, it is recommended to adopt a longitudinal time-series design, conduct multicenter sampling across different regions. Additionally, in terms of variable selection, multiple related factors such as organizational atmosphere, mindfulness, and leadership style should be included. Meanwhile, work procrastination, as a manifestation of the nurses’ state, not only plays the role of an outcome variable but may also serve as an antecedent variable for other factors. Thus, it is recommended to enrich and integrate the research on nurses’ work procrastination from multiple perspectives, actively exploring its role as a mediator or antecedent variable, thereby enhancing the theoretical and practical research on clinical nurses’ work procrastination.

## 6 Conclusion

In this study, we conducted a latent profile analysis of clinical nurses’ procrastination in central China through an individual-centered approach and explored the correlations between smartphone addiction, demographic differences, and the profiles of procrastination. Ultimately, we found that clinical nurses are at a moderately low-level procrastination in their work. Their procrastination behavior can be classified into three subgroups: low procrastination, mid-low procrastination, and mid-high procrastination. At the same time, surgical nurses or clinical nurses with intermediate titles exhibit higher levels of procrastination. Additionally, nurses who are addicted to their phones, have higher monthly incomes, work in tense departmental atmospheres, and face difficulties in career advancement are more prone to work procrastination. Conversely, hospitals with strict management and head nurses show lower levels of procrastination. These findings confirm the hypotheses H1 to H3 proposed in this study. Theoretically, our research employed the Latent Profile Analysis (LPA) mixed modeling approach to reveal insights that could not captured by the variable-centered analysis methods, enriching the study of clinical nurses’ procrastination. Secondarily, this study systematically analyzes nurse work procrastination by incorporating mobile phone addiction and relevant demographic variables, offering a deeper understanding of the causal mechanisms of procrastination and further enriching the existing theoretical framework. Additionally, by exploring the relationship between clinical nurses’ mobile phone usage and work procrastination, the study unveils the role of mobile phone addiction in procrastination behaviors, thereby providing theoretical support for addiction psychology and intervention strategies. In practical terms, this research offers a novel perspective and approach for studying work procrastination among clinical nurses, facilitating the translation of relevant theories into practice. At the individual level, it can aid clinical nurses in recognizing their own procrastination status and supports their self-regulation. At the organizational level, it can assist hospital managers in comprehending the causes and impacts of work procrastination among clinical nurses, thereby enabling the development of targeted improvement strategies and training programs. This, in turn, enhances nursing staff efficiency and professional standards, and improves the quality of nursing services. Therefore, it is suggested that nursing managers actively pay attention to the procrastination status of clinical nurses, particularly focusing on providing psychological guidance for nurses with moderate and higher levels of procrastination. Tailored training or interventions based on individual circumstances are recommended. Clinical nurses are also encouraged to enhance self-control and time management skills, optimize their career development strategies, and thereby improve work efficiency and reduce procrastination.

## Data availability statement

The original contributions presented in the study are included in the article/Supplementary material, further inquiries can be directed to the corresponding author.

## Ethics statement

The studies involving humans were approved by People’s Hospital of Henan University of Traditional Chinese Medicine. The studies were conducted in accordance with the local legislation and institutional requirements. The participants provided their written informed consent to participate in this study.

## Author contributions

HX: Methodology, Writing – original draft, Writing – review & editing. SJ: Data curation, Investigation, Writing – review & editing. XSo: Data curation, Investigation, Writing – review & editing. FZ: Conceptualization, Writing – review & editing. XL: Investigation, Supervision, Writing – review & editing. XSi: Writing – original draft, Writing – review & editing.

## Footnotes

^1^ https://www.wjx.cn

## References

[ref1] Babaie M. Farahani A. S. Nourian M. Hosseini M. Mohammadi A. (2022). Assessment of procrastination in providing nursing care among Iranian nursing staff. BMC Nurs. 21:343. doi: 10.1186/s12912-022-01132-5, PMID: 36471310 PMC9724313

[ref2] Basirimoghadam M. Rafii F. Ebadi A. (2020). Self-rated health and general procrastination in nurses: a cross-sectional study. Pan Afr. Med. J. 36:254. doi: 10.11604/pamj.2020.36.254.23720, PMID: 33014250 PMC7519796

[ref3] Basirimoghadam M. Rafii F. Ebadi A. (2023). Development and psychometric evaluation of nurses' health-related procrastination scale. Heliyon 9:e18145. doi: 10.1016/j.heliyon.2023.e18145, PMID: 37519648 PMC10372369

[ref4] Bautista J. R. (2020). Policy recommendations on nurses' use of smartphones in the Philippines. Int. J. Med. Inform. 142:104250. doi: 10.1016/j.ijmedinf.2020.104250, PMID: 32828988

[ref5] Benites-Zapata V. A. Jiménez-Torres V. E. Ayala-Roldán M. P. (2021). Problematic smartphone use is associated with de Quervain's tenosynovitis symptomatology among young adults. Musculoskelet. Sci. Pract. 53:102356. doi: 10.1016/j.msksp.2021.102356, PMID: 33667881

[ref6] Brand M. Wegmann E. Stark R. Müller A. Wölfling K. Robbins T. W.. (2019). The interaction of person-affect-cognition-execution (I-PACE) model for addictive behaviors: update, generalization to addictive behaviors beyond internet-use disorders, and specification of the process character of addictive behaviors. Neurosci. Biobehav. Rev. 104, 1–10. doi: 10.1016/j.neubiorev.2019.06.032, PMID: 31247240

[ref7] Buneviciene I. Bunevicius A. (2021). Prevalence of internet addiction in healthcare professionals: systematic review and meta-analysis. Int. J. Soc. Psychiatry 67, 483–491. doi: 10.1177/0020764020959093, PMID: 32962501

[ref8] Chen Z. Y. Zhang R. Xu T. Yang Y. Q. Wang J. Y. Feng T. Y. (2020). Emotional attitudes towards procrastination in people: a large-scale sentiment-focused crawling analysis. Comput. Hum. Behav. 110:106391. doi: 10.1016/j.chb.2020.106391

[ref9] Cuccia A. F. Peterson C. Melnyk B. M. Boston-Leary K. (2022). Trends in mental health indicators among nurses participating in healthy nurse, healthy nation from 2017 to 2021. Worldviews Evid.-Based Nurs. 19, 352–358. doi: 10.1111/wvn.12601 35934812

[ref10] Dai C. Qiu H. Huang Q. Hu P. Hong X. Tu J.. (2019). The effect of night shift on sleep quality and depressive symptoms among Chinese nurses. Neuropsychiatr. Dis. Treat. 15, 435–440. doi: 10.2147/ndt.S190689 30799922 PMC6369837

[ref11] Darnai G. Perlaki G. Zsidó A. N. Inhóf O. Orsi G. Horváth R.. (2019). Internet addiction and functional brain networks: task-related fMRI study. Sci. Rep. 9:15777. doi: 10.1038/s41598-019-52296-1, PMID: 31673061 PMC6823489

[ref12] De Jong A. Donelle L. Kerr M. (2020). Nurses' use of personal smartphone Technology in the Workplace: scoping review. JMIR Mhealth Uhealth 8:e18774. doi: 10.2196/18774, PMID: 33242012 PMC7728531

[ref13] Elhai J. D. Dvorak R. D. Levine J. C. Hall B. J. (2017). Problematic smartphone use: a conceptual overview and systematic review of relations with anxiety and depression psychopathology. J. Affect. Disord. 207, 251–259. doi: 10.1016/j.jad.2016.08.030 27736736

[ref14] Ferramosca F. M. P. De Maria M. Ivziku D. Raffaele B. Lommi M. Tolentino Diaz M. Y.. (2023). Nurses' Organization of work and its relation to workload in medical surgical units: a cross-sectional observational multi-center study. Healthcare 11:156. doi: 10.3390/healthcare11020156, PMID: 36673524 PMC9858832

[ref15] Genrich M. Worringer B. Angerer P. Müller A. (2020). Hospital medical and nursing Managers' perspectives on health-related work design interventions. A Qualitative Study. Front Psychol 11:869. doi: 10.3389/fpsyg.2020.00869, PMID: 32431651 PMC7214727

[ref16] Hao C. Zhu L. Zhang S. Rong S. Zhang Y. Ye J.. (2020). Serial multiple mediation of professional identity, and psychological Capital in the Relationship between Work-Related Stress and Work-Related Well-Being of ICU nurses in China: a cross-sectional questionnaire survey. Front. Psychol. 11:535634. doi: 10.3389/fpsyg.2020.535634, PMID: 33414737 PMC7782242

[ref17] Hen M. Goroshit M. Viengarten S. (2021). How decisional and general procrastination relate to procrastination at work: an investigation of office and non-office workers. Personal. Individ. Differ. 172:110581. doi: 10.1016/j.paid.2020.110581

[ref18] Hølge-Hazelton B. Berthelsen C. B. (2020). Why do nurses stay? A positive deviance study of nurse turnover. Eur. J. Pub. Health 30:629. doi: 10.1093/eurpub/ckaa166.629

[ref19] James R. J. E. Dixon G. Dragomir M. G. Thirlwell E. Hitcham L. (2023). Understanding the construction of 'behavior' in smartphone addiction: a scoping review. Addict. Behav. 137:107503. doi: 10.1016/j.addbeh.2022.107503, PMID: 36228362

[ref20] Kalamara E. Richardson C. (2022). Using latent profile analysis to understand burnout in a sample of Greek teachers. Int. Arch. Occup. Environ. Health 95, 141–152. doi: 10.1007/s00420-021-01780-1, PMID: 34636978

[ref21] Karadağ E. Tosuntaş Ş. B. Erzen E. Duru P. Bostan N. Şahin B. M.. (2015). Determinants of phubbing, which is the sum of many virtual addictions: a structural equation model. J. Behav. Addict. 4, 60–74. doi: 10.1556/2006.4.2015.005, PMID: 26014669 PMC4500886

[ref22] Leung L. (2008). Linking psychological attributes to addiction and improper use of the mobile phone among adolescents in Hong Kong. J. Child. Media 2, 93–113. doi: 10.1080/17482790802078565

[ref23] Lian S. Bai X. Zhu X. Sun X. Zhou Z. (2022). How and for whom is Mobile phone addiction associated with mind wandering: the mediating role of fatigue and moderating role of rumination. Int. J. Environ. Res. Public Health 19:886. doi: 10.3390/ijerph192315886, PMID: 36497958 PMC9741139

[ref24] Lim V. K. G. Teo T. S. H. (2024). Cyberloafing: a review and research agenda. Appl. Psychol. 73, 441–484. doi: 10.1111/apps.12452

[ref25] Lin C. Y. Ratan Z. A. Pakpour A. H. (2023). Collection of smartphone and internet addiction. BMC Psychiatry 23:427. doi: 10.1186/s12888-023-04915-5, PMID: 37316810 PMC10265856

[ref26] Liu Y. Aungsuroch Y. (2019). Work stress, perceived social support, self-efficacy and burnout among Chinese registered nurses. J. Nurs. Manag. 27, 1445–1453. doi: 10.1111/jonm.12828 31306524

[ref27] Lu H. Zhao Y. While A. (2019). Job satisfaction among hospital nurses: a literature review. Int. J. Nurs. Stud. 94, 21–31. doi: 10.1016/j.ijnurstu.2019.01.011 30928718

[ref28] Luk T. T. Wang M. P. Shen C. Wan A. Chau P. H. Oliffe J.. (2018). Short version of the smartphone addiction scale in Chinese adults: psychometric properties, sociodemographic, and health behavioral correlates. J. Behav. Addict. 7, 1157–1165. doi: 10.1556/2006.7.2018.105, PMID: 30418073 PMC6376375

[ref29] Ma H. Zou J. M. Zhong Y. He J. Q. (2021). The influence of mobile phone addiction and work procrastination on burnout among newly graduated Chinese nurses. Perspect. Psychiatr. Care 57, 1798–1805. doi: 10.1111/ppc.12752, PMID: 33651417

[ref30] Mcbride D. L. Levasseur S. A. Li D. (2015). Non-work-related use of personal mobile phones by hospital registered nurses. JMIR Mhealth Uhealth 3:e3. doi: 10.2196/mhealth.4001, PMID: 25586982 PMC4319148

[ref31] Metin U. B. Peeters M. C. W. Taris T. W. (2018). Correlates of procrastination and performance at work: the role of having "good fit". J. Prev. Interv. Community 46, 228–244. doi: 10.1080/10852352.2018.1470187, PMID: 30024357

[ref32] Metin U. B. Tanis T. W. Peeters M. C. W. (2016). Measuring procrastination at work and its associated workplace aspects. Personal. Individ. Differ. 101, 254–263. doi: 10.1016/j.paid.2016.06.006

[ref33] Morse V. Warshawsky N. E. (2021). Nurse leader competencies: today and tomorrow. Nurs. Adm. Q. 45, 65–70. doi: 10.1097/NAQ.0000000000000453 33259373

[ref34] Nylund-Gibson K. Grimm R. P. Masyn K. E. (2019). Prediction from latent classes: a demonstration of different approaches to include distal outcomes in mixture models. Struct. Equ. Model. Multidiscip. J. 26, 967–985. doi: 10.1080/10705511.2019.1590146

[ref35] O'connor S. Chu C. H. Thilo F. Lee J. J. Mather C. Topaz M. (2020). Professionalism in a digital and mobile world: a way forward for nursing. J. Adv. Nurs. 76, 4–6. doi: 10.1111/jan.14224 31588582

[ref36] Olson J. A. Sandra D. A. Colucci É. Al Bikaii A. Chmoulevitch D. Nahas J.. (2022). Smartphone addiction is increasing across the world: a meta-analysis of 24 countries. Comput. Hum. Behav. 129:107138. doi: 10.1016/j.chb.2021.107138

[ref37] Olson J. A. Sandra D. A. Veissière S. P. L. Langer E. J. (2023). Sex, age, and smartphone addiction across 41 countries. Int. J. Ment. Heal. Addict. 1–9. doi: 10.1007/s11469-023-01146-3

[ref38] Osorio-Molina C. Martos-Cabrera M. B. Membrive-Jiménez M. J. Vargas-Roman K. Suleiman-Martos N. Ortega-Campos E.. (2021). Smartphone addiction, risk factors and its adverse effects in nursing students: a systematic review and meta-analysis. Nurse Educ. Today 98:104741. doi: 10.1016/j.nedt.2020.104741 33485161

[ref39] Panova T. Carbonell X. (2018). Is smartphone addiction really an addiction? J. Behav. Addict. 7, 252–259. doi: 10.1556/2006.7.2018.49, PMID: 29895183 PMC6174603

[ref40] Podsakoff P. M. Organ D. W. (1986). Self-reports in organizational research – problems and prospects. J. Manag. 12, 531–544. doi: 10.1177/014920638601200408

[ref41] Ratan Z. A. Parrish A. M. Zaman S. B. Alotaibi M. S. Hosseinzadeh H. (2021). Smartphone addiction and associated health outcomes in adult populations: a systematic review. Int. J. Environ. Res. Public Health 18:257. doi: 10.3390/ijerph182212257, PMID: 34832011 PMC8622754

[ref42] Rebetez M. M. L. Rochat L. Barsics C. Van Der Linden M. (2018). Procrastination as a self-regulation failure: the role of impulsivity and intrusive thoughts. Psychol. Rep. 121, 26–41. doi: 10.1177/0033294117720695 28776482

[ref43] Rezaei B. Yarmohammadian M. H. Ardakani H. M. (2017). Te relationship between nurse Managers' leadership styles and procrastination in nursing staff in Isfahan social welfare hospitals. Sci. J. Hamedan Nurs. Midwifery Fac. 25, 60–68. doi: 10.21859/nmj-25018

[ref44] Riley R. D. Ensor J. Snell K. I. E. Harrell F. E. Jr. Martin G. P. Reitsma J. B.. (2020). Calculating the sample size required for developing a clinical prediction model. BMJ 368:m441. doi: 10.1136/bmj.m441 32188600

[ref45] Salvage J. White J. (2019). Nursing leadership and health policy: everybody's business. Int. Nurs. Rev. 66, 147–150. doi: 10.1111/inr.12523, PMID: 31124127

[ref46] Schroeder G. L. Heckler W. Francisco R. Barbosa J. L. V. (2022). Problematic smartphone use on mental health: a systematic mapping study and taxonomy. Behav. Inform. Technol. 42, 2808–2831. doi: 10.1080/0144929x.2022.2149422

[ref47] Schunk D. H. Dibenedetto M. K. (2020). Motivation and social cognitive theory. Contemp. Educ. Psychol. 60:101832. doi: 10.1016/j.cedpsych.2019.101832

[ref48] Si X. Xue H. Song X. Liu X. Zhang F. (2023). The relationship between ethical leadership and nurse well-being: the mediating role of workplace mindfulness. J. Adv. Nurs. 79, 4008–4021. doi: 10.1111/jan.15719, PMID: 37226654

[ref49] Sirois F. M. Stride C. B. Pychyl T. A. (2023). Procrastination and health: a longitudinal test of the roles of stress and health behaviours. Br. J. Health Psychol. 28, 860–875. doi: 10.1111/bjhp.12658, PMID: 36919887

[ref50] Spurk D. Hirschi A. Wang M. Valero D. Kauffeld S. (2020). Latent profile analysis: a review and “how to” guide of its application within vocational behavior research. J. Vocat. Behav. 120:103445. doi: 10.1016/j.jvb.2020.103445

[ref51] Steel P. (2007). The nature of procrastination: a meta-analytic and theoretical review of quintessential self-regulatory failure. Psychol. Bull. 133, 65–94. doi: 10.1037/0033-2909.133.1.65, PMID: 17201571

[ref52] Van Den Broeck A. Ferris D. L. Chang C. H. Rosen C. C. (2016). A review of self-determination Theory's basic psychological needs at work. J. Manag. 42, 1195–1229. doi: 10.1177/0149206316632058

[ref53] Volkow N. D. Wise R. A. Baler R. (2017). The dopamine motive system: implications for drug and food addiction. Nat. Rev. Neurosci. 18, 741–752. doi: 10.1038/nrn.2017.130, PMID: 29142296

[ref54] Wang J. Li C. Meng X. Liu D. (2021). Validation of the Chinese version of the procrastination at work scale. Front. Psychol. 12:726595. doi: 10.3389/fpsyg.2021.726595, PMID: 34603147 PMC8481865

[ref55] Wang Q. Q. Lv W. J. Qian R. L. Zhang Y. H. (2019). Job burnout and quality of working life among Chinese nurses: a cross-sectional study. J. Nurs. Manag. 27, 1835–1844. doi: 10.1111/jonm.12884, PMID: 31571326

[ref56] Wei L. Guo Z. Zhang X. Niu Y. Wang X. Ma L.. (2023). Mental health and job stress of nurses in surgical system: what should we care. BMC Psychiatry 23:871. doi: 10.1186/s12888-023-05336-0, PMID: 37996803 PMC10666426

[ref57] Yinghao Z. Dan Z. Qi L. Yu W. Xiaoying W. Ao F.. (2023). A cross-sectional study of clinical emergency department nurses' occupational stress, job involvement and team resilience. Int. Emerg. Nurs. 69:101299. doi: 10.1016/j.ienj.2023.101299 37269628

[ref58] Yu H. (2024). Why do people use Metaverse? A uses and gratification theory perspective. Telematics Inform. 89:102110. doi: 10.1016/j.tele.2024.102110

[ref59] Zhang S. Liu P. Feng T. (2019). To do it now or later: the cognitive mechanisms and neural substrates underlying procrastination. Wiley Interdiscip. Rev. Cogn. Sci. 10:e1492. doi: 10.1002/wcs.1492, PMID: 30638308

